# Sulfonated 1,3-bis­(4-pyrid­yl)propane

**DOI:** 10.1107/S1600536811018563

**Published:** 2011-05-28

**Authors:** Ore Kuyinu, Andrew P. Purdy, Ray J. Butcher

**Affiliations:** a Chemistry Division, Code 6120 Naval Research Laboratory, 4555 Overlook Avenue SW, Washington, DC 20375, USA; b Department of Chemistry, Howard University, 525 College Street NW, Washington, DC 20059, USA

## Abstract

In the title compound, 4-[3-(3-sulfonato­pyridin-1-ium-4-yl)prop­yl]pyridin-1-ium-3-sulfonate, C_13_H_14_N_2_O_6_S_2_, the mol­ecule is zwitterionic, with the sulfonic acid proton transfered to the basic pyridine N atom. Also, the structure adopts a butterfly-like conformation with the sulfonate groups on opposite sides of the ‘wings’. The dihedral angle between the two pyridinium rings is 83.56 (7)°, and this results in the mol­ecule having a chiral conformation and packing. There is strong inter­molecular hydrogen bonding between the pyridinium H and sulfonate O atoms of adjoining mol­ecules. In addition, there are weaker inter­molecular C—H⋯O inter­actions.

## Related literature

For zwiterionic polymers, see: Estrin & Entelis (1974 ▶); Sundaram *et al.* (2010 ▶). For 1,3-bis­(4-pyrid­yl)propane ligands, see: Chen *et al.* (2010 ▶); Correa *et al.* (2010 ▶); Sun *et al.* (2010 ▶); Zheng *et al.* (2010 ▶). For sulfonation of pyridine rings, see: McElvain & Goese (1943 ▶).
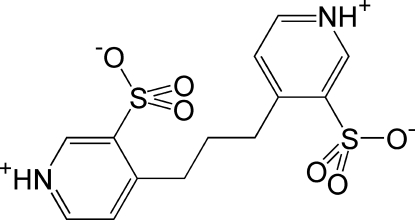


## Experimental

### 

#### Crystal data

C_13_H_14_N_2_O_6_S_2_
*M* *_r_* = 358.38
Orthorhombic, 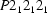

*a* = 9.7132 (2) Å
*b* = 11.2624 (2) Å
*c* = 13.5369 (2) Å
*V* = 1480.85 (5) Å^3^
*Z* = 4
Cu *K*α radiation
μ = 3.59 mm^−1^
*T* = 295 K
0.77 × 0.25 × 0.19 mm

#### Data collection

Oxford Diffraction Xcalibur Ruby Gemini diffractometer
Absorption correction: analytical [*CrysAlis PRO* (Oxford Diffraction, 2007 ▶), based on expressions derived by Clark & Reid (1995 ▶)] *T*_min_ = 0.290, *T*_max_ = 0.626
3960 measured reflections
2714 independent reflections
2593 reflections with *I* > 2σ(*I*)
*R*_int_ = 0.021

#### Refinement

*R*[*F* ^2^ > 2σ(*F* ^2^)] = 0.038
*wR*(*F* ^2^) = 0.106
*S* = 1.06
2714 reflections
208 parameters
12 restraints
H-atom parameters constrained
Δρ_max_ = 0.51 e Å^−3^
Δρ_min_ = −0.52 e Å^−3^
Absolute structure: Flack (1983 ▶), 899 Friedel pairs
Flack parameter: 0.07 (2)

### 

Data collection: *CrysAlis PRO* (Oxford Diffraction, 2007 ▶); cell refinement: *CrysAlis PRO*; data reduction: *CrysAlis PRO*; program(s) used to solve structure: *SHELXS97* (Sheldrick, 2008 ▶); program(s) used to refine structure: *SHELXL97* (Sheldrick, 2008 ▶); molecular graphics: *SHELXTL* (Sheldrick, 2008 ▶); software used to prepare material for publication: *SHELXTL*.

## Supplementary Material

Crystal structure: contains datablocks I, global. DOI: 10.1107/S1600536811018563/fl2337sup1.cif

Structure factors: contains datablocks I. DOI: 10.1107/S1600536811018563/fl2337Isup2.hkl

Supplementary material file. DOI: 10.1107/S1600536811018563/fl2337Isup3.cml

Additional supplementary materials:  crystallographic information; 3D view; checkCIF report

## Figures and Tables

**Table 1:** Hydrogen-bond geometry (Å, °)

*D*—H⋯*A*	*D*—H	H⋯*A*	*D*⋯*A*	*D*—H⋯*A*
N1*A*—H1*AA*⋯O3*A*^i^	0.86	1.88	2.706 (3)	159
N1*A*—H1*AA*⋯S1^i^	0.86	2.79	3.633 (3)	165
N1*B*—H1*BA*⋯O2*B*^ii^	0.86	1.85	2.710 (3)	176
N1*B*—H1*BA*⋯S2^ii^	0.86	2.85	3.655 (2)	158
C3*B*—H3*BA*⋯O1*A*^iii^	0.93	2.44	3.008 (3)	119
C4*B*—H4*BA*⋯O2*A*^iv^	0.93	2.50	3.035 (3)	117
C5*B*—H5*BA*⋯O1*B*^v^	0.93	2.58	3.416 (4)	150

Symmetry codes: (i) 
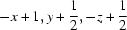
; (ii) 
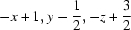
; (iii) 
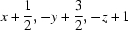
; (iv) 
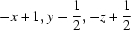
; (v) 
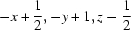
.

**Fig. 1.**
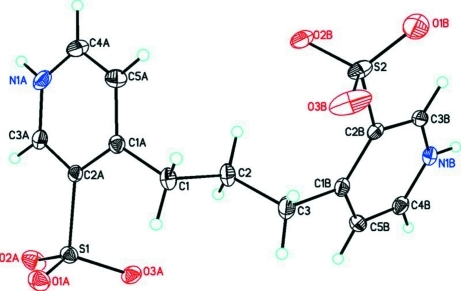
Diagram of C13H14N2O6S2 illustrating the atom numbering scheme used. Thermal ellipsoids are at the 30% probability level.

**Fig. 2.**
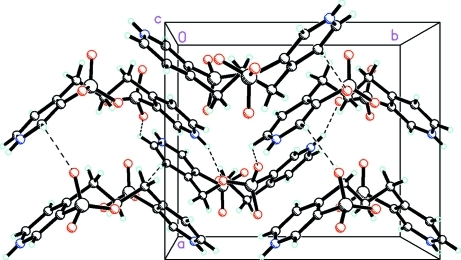
The molecular packing for C13H14N2O6S2 viewed down the c axis showing the hydrogen bonds as dashed lines.

## supplementary crystallographic information

### Comment

From the titled compound C_13_H_14_N_2_O_6_S_2_ we can see that the sulfonatation of 1,3-bis(4-pyridyl)propane occurs in the *meta* position under mercury catalysis, the same as when pyridine is sulfonated under similar conditions (McElvain & Goese, 1943). From recent studies, 1,3-bis(4-pyridyl)propane ligands have been shown (Chen *et al.*, 2010; Correa *et al.*, 2010; Sun *et al.*, 2010; Zheng *et al.*, 2010) to be very flexible, and this has been taken advantage of in supramolecular chemistry. Our interest was to link the sulfonated pyridines into a zwitterionic polymer. (Estrin & Entelis, 1974; Sundaram *et al.*, 2010). Polymer zwitterions are very advantageous in the sense that their distinct polar ends have high electric dipoles, and can readily reorient to an applied electric field if the chain is flexible.

In view of the interest in a flexible zwitterionic polymer backbone, the starting material, 1,3-bis(4-pyridyl)propane was sulfonated. The structure of this derivative is reported here. The structure shows that both pyridine rings have been sulfonated in the 3-position. As is to be expected for a moiety containing both acidic and basic substituents, the molecule is zwitterionic, with the sulfonic acid proton transfered to the basic pyridine N. The structure has adopted a "butterfly-like" conformation with the sulfonate groups on opposite sides of the "wings". The dihedral angle of 83.56 (7)° between the two pyridinium rings is shown in Figure 2. This has resulted in the molecule being chiral in the solid state even though it is not chiral in solution. There is strong intermolecular hydrogen bonding between the pyridinium H and sulfonate O atoms of adjoining molecules. In addition there are weaker intermolecular C—H···O interactions.

### Experimental

The compound was prepared by adding a mercury catalyst (0.05 g) to 0.33 g of 1,3-bis(4-pyridyl)propane dissolved in 3.24 g of fuming sulfuric acid (oleum). The reaction mixture was placed in a quartz tube that was sealed under vacuum with a fill factor of 10/15.4 cm. The quartz tube was then placed into a pressurized hydrothermal vessel that was set in a furnace at a temperature of 245°C. The pressure vessel attained an internal temperature of 204°C, and the reaction continued for 5 days. After cooling, the quartz tube was removed from the chamber behind a blast shield in a fume hood, and was frozen with liquid nitrogen before opening. The liquid was then poured into an Erlenmeyer flask containing about 10 ml of triply distilled water and was allowed to stand. Crystals of the titled compound slowly appeared over a month at which point they were washed with alcohol and allowed to air dry on top of the oven at about 50°C. Approximately 0.15 g was isolated (25%). MP: dec 320. NMR: (D_2_O/DSS): ^1^H, 2.28 (CH_2_, 2H), 3.44 (CH_2_, 4H), 8.13 (py, 2H), 8.79 (py, 2H), 9.13 (py, 2H); ^13^C 31.75 (CH_2_, 1 C), 35.39 (CH_2_, 2 C), 131.97, 142.50, 144.09, 145.02, 164.74 (py).

### Refinement

H atoms were placed in geometrically idealized positions and constrained to ride on their parent atoms with a C—H distance of 0.93 and 0.97 Å *U*_iso_(H) = 1.2*U*_eq_(C). The H atoms attached to N were idealized with an N–H distance of 0.86 Å.

### Crystal data

**Table:**

C_13_H_14_N_2_O_6_S_2_	*D*_x_ = 1.607 Mg m^−^^3^
*M**_r_* = 358.38	Cu *K*α radiation, λ = 1.54178 Å
Orthorhombic, *P*2_1_2_1_2_1_	Cell parameters from 3437 reflections
*a* = 9.7132 (2) Å	θ = 4.6–77.4°
*b* = 11.2624 (2) Å	µ = 3.59 mm^−^^1^
*c* = 13.5369 (2) Å	*T* = 295 K
*V* = 1480.85 (5) Å^3^	Needle, pale yellow
*Z* = 4	0.77 × 0.25 × 0.19 mm
*F*(000) = 744	

### Data collection

**Table:**

Oxford Diffraction Xcalibur Ruby Gemini diffractometer	2714 independent reflections
Radiation source: Enhance (Cu) X-ray Source	2593 reflections with *I* > 2σ(*I*)
graphite	*R*_int_ = 0.021
Detector resolution: 10.5081 pixels mm^-1^	θ_max_ = 77.6°, θ_min_ = 5.1°
ω scans	*h* = −8→12
Absorption correction: analytical [*CrysAlis PRO* (Oxford Diffraction, 2007), based on expressions derived by Clark & Reid (1995)]	*k* = −11→14
*T*_min_ = 0.290, *T*_max_ = 0.626	*l* = −16→13
3960 measured reflections	

### Refinement

**Table:**

Refinement on *F*^2^	Secondary atom site location: difference Fourier map
Least-squares matrix: full	Hydrogen site location: inferred from neighbouring sites
*R*[*F*^2^ > 2σ(*F*^2^)] = 0.038	H-atom parameters constrained
*wR*(*F*^2^) = 0.106	*w* = 1/[σ^2^(*F*_o_^2^) + (0.0719*P*)^2^ + 0.3816*P*] where *P* = (*F*_o_^2^ + 2*F*_c_^2^)/3
*S* = 1.06	(Δ/σ)_max_ < 0.001
2714 reflections	Δρ_max_ = 0.51 e Å^−^^3^
208 parameters	Δρ_min_ = −0.52 e Å^−^^3^
12 restraints	Absolute structure: Flack (1983), 899 Friedel pairs
Primary atom site location: structure-invariant direct methods	Flack parameter: 0.07 (2)

### Special details

**Table:**

Experimental. CrysAlis Pro (Oxford Diffraction, 2007) Analytical numeric absorption correction using a multifaceted crystal model, based on expressions derived by Clark & Reid (1995)
Geometry. All e.s.d.'s (except the e.s.d. in the dihedral angle between two l.s. planes) are estimated using the full covariance matrix. The cell e.s.d.'s are taken into account individually in the estimation of e.s.d.'s in distances, angles and torsion angles; correlations between e.s.d.'s in cell parameters are only used when they are defined by crystal symmetry. An approximate (isotropic) treatment of cell e.s.d.'s is used for estimating e.s.d.'s involving l.s. planes.
Refinement. Refinement of *F*^2^ against ALL reflections. The weighted *R*-factor *wR* and goodness of fit *S* are based on *F*^2^, conventional *R*-factors *R* are based on *F*, with *F* set to zero for negative *F*^2^. The threshold expression of *F*^2^ > σ(*F*^2^) is used only for calculating *R*-factors(gt) *etc*. and is not relevant to the choice of reflections for refinement. *R*-factors based on *F*^2^ are statistically about twice as large as those based on *F*, and *R*- factors based on ALL data will be even larger.

### Fractional atomic coordinates and isotropic or equivalent isotropic displacement parameters (Å^2^)

**Table:**

	*x*	*y*	*z*	*U*_iso_*/*U*_eq_	
S1	0.28273 (7)	0.82506 (6)	0.18353 (4)	0.03490 (17)	
S2	0.25484 (7)	0.69702 (6)	0.73312 (6)	0.0450 (2)	
O1A	0.1398 (2)	0.8521 (2)	0.16464 (17)	0.0501 (6)	
O2A	0.3744 (3)	0.8523 (3)	0.10328 (15)	0.0587 (7)	
O3A	0.3027 (2)	0.70553 (17)	0.22295 (16)	0.0444 (5)	
O1B	0.2815 (3)	0.6781 (2)	0.8361 (2)	0.0741 (8)	
O2B	0.3281 (3)	0.80026 (19)	0.69595 (18)	0.0521 (5)	
O3B	0.1119 (3)	0.6967 (3)	0.7050 (3)	0.0883 (11)	
N1A	0.4665 (3)	1.0862 (2)	0.3287 (2)	0.0478 (6)	
H1AA	0.5265	1.1394	0.3135	0.057*	
N1B	0.4929 (2)	0.4168 (2)	0.68491 (18)	0.0356 (5)	
H1BA	0.5502	0.3772	0.7204	0.043*	
C1	0.1965 (3)	0.8097 (3)	0.41421 (19)	0.0386 (6)	
H1A	0.1477	0.7738	0.3593	0.046*	
H1B	0.1293	0.8374	0.4620	0.046*	
C2	0.2932 (3)	0.7187 (3)	0.4620 (2)	0.0424 (6)	
H2A	0.3445	0.7570	0.5147	0.051*	
H2B	0.3588	0.6912	0.4131	0.051*	
C3	0.2151 (3)	0.6114 (3)	0.5045 (2)	0.0479 (7)	
H3A	0.1350	0.6384	0.5409	0.057*	
H3B	0.1841	0.5605	0.4512	0.057*	
C1A	0.2832 (3)	0.9119 (2)	0.37844 (17)	0.0326 (5)	
C2A	0.3323 (3)	0.9228 (2)	0.28185 (18)	0.0301 (5)	
C3A	0.4240 (3)	1.0107 (2)	0.2592 (2)	0.0391 (6)	
H3AA	0.4569	1.0178	0.1950	0.047*	
C4A	0.4185 (4)	1.0818 (3)	0.4211 (3)	0.0553 (9)	
H4AA	0.4473	1.1374	0.4675	0.066*	
C5A	0.3276 (4)	0.9959 (3)	0.4470 (2)	0.0486 (7)	
H5AA	0.2945	0.9929	0.5114	0.058*	
C1B	0.3094 (3)	0.5428 (2)	0.57229 (19)	0.0356 (5)	
C2B	0.3329 (3)	0.5730 (2)	0.67150 (19)	0.0310 (5)	
C3B	0.4251 (3)	0.5075 (2)	0.72588 (19)	0.0338 (5)	
H3BA	0.4405	0.5264	0.7918	0.041*	
C4B	0.4747 (3)	0.3857 (2)	0.5911 (2)	0.0401 (6)	
H4BA	0.5246	0.3229	0.5645	0.048*	
C5B	0.3826 (3)	0.4462 (3)	0.53385 (19)	0.0414 (6)	
H5BA	0.3684	0.4230	0.4687	0.050*	

### Atomic displacement parameters (Å^2^)

**Table:**

	*U*^11^	*U*^22^	*U*^33^	*U*^12^	*U*^13^	*U*^23^
S1	0.0363 (3)	0.0368 (3)	0.0316 (3)	0.0032 (3)	−0.0007 (2)	−0.0034 (2)
S2	0.0395 (4)	0.0328 (3)	0.0627 (4)	−0.0030 (3)	0.0108 (3)	−0.0146 (3)
O1A	0.0454 (12)	0.0517 (13)	0.0531 (12)	0.0097 (10)	−0.0129 (9)	−0.0057 (10)
O2A	0.0598 (14)	0.0826 (18)	0.0336 (9)	−0.0084 (14)	0.0095 (10)	−0.0077 (11)
O3A	0.0485 (11)	0.0315 (9)	0.0532 (11)	0.0056 (9)	−0.0039 (9)	−0.0078 (8)
O1B	0.102 (2)	0.0568 (14)	0.0640 (14)	0.0053 (16)	0.0363 (15)	−0.0128 (12)
O2B	0.0640 (14)	0.0320 (10)	0.0604 (13)	−0.0069 (10)	−0.0061 (11)	−0.0096 (9)
O3B	0.0386 (12)	0.0712 (18)	0.155 (3)	0.0011 (13)	0.0049 (16)	−0.055 (2)
N1A	0.0484 (14)	0.0281 (11)	0.0669 (17)	−0.0097 (10)	−0.0099 (13)	0.0047 (11)
N1B	0.0359 (11)	0.0312 (10)	0.0396 (11)	0.0001 (9)	0.0002 (9)	0.0083 (9)
C1	0.0378 (13)	0.0425 (14)	0.0354 (11)	0.0022 (12)	0.0038 (10)	0.0110 (11)
C2	0.0405 (14)	0.0468 (14)	0.0401 (13)	−0.0009 (13)	0.0002 (12)	0.0174 (11)
C3	0.0466 (16)	0.0459 (15)	0.0511 (15)	−0.0080 (14)	−0.0070 (14)	0.0181 (13)
C1A	0.0353 (12)	0.0307 (11)	0.0319 (11)	0.0061 (11)	0.0011 (10)	0.0021 (9)
C2A	0.0327 (11)	0.0260 (10)	0.0314 (10)	0.0043 (10)	−0.0011 (9)	0.0024 (9)
C3A	0.0415 (14)	0.0333 (12)	0.0425 (14)	−0.0003 (11)	−0.0002 (11)	0.0086 (11)
C4A	0.071 (2)	0.0360 (15)	0.0585 (18)	−0.0021 (15)	−0.0121 (17)	−0.0129 (14)
C5A	0.0600 (19)	0.0457 (16)	0.0401 (14)	0.0031 (15)	0.0010 (13)	−0.0098 (12)
C1B	0.0388 (13)	0.0312 (12)	0.0368 (12)	−0.0063 (11)	0.0020 (11)	0.0079 (10)
C2B	0.0337 (12)	0.0230 (10)	0.0363 (12)	−0.0032 (9)	0.0067 (10)	−0.0013 (9)
C3B	0.0383 (12)	0.0332 (12)	0.0299 (11)	−0.0069 (10)	0.0025 (10)	−0.0008 (10)
C4B	0.0488 (15)	0.0286 (12)	0.0428 (14)	0.0014 (12)	0.0105 (12)	−0.0010 (11)
C5B	0.0557 (16)	0.0412 (14)	0.0272 (11)	−0.0061 (14)	0.0046 (11)	−0.0023 (11)

### Geometric parameters (Å, °)

**Table:**

S1—O2A	1.438 (2)	C2—H2A	0.9700
S1—O1A	1.444 (2)	C2—H2B	0.9700
S1—O3A	1.461 (2)	C3—C1B	1.509 (4)
S1—C2A	1.793 (3)	C3—H3A	0.9700
S2—O1B	1.433 (3)	C3—H3B	0.9700
S2—O3B	1.439 (3)	C1A—C5A	1.394 (4)
S2—O2B	1.453 (2)	C1A—C2A	1.397 (3)
S2—C2B	1.795 (2)	C2A—C3A	1.366 (4)
N1A—C3A	1.334 (4)	C3A—H3AA	0.9300
N1A—C4A	1.337 (5)	C4A—C5A	1.356 (5)
N1A—H1AA	0.8600	C4A—H4AA	0.9300
N1B—C4B	1.329 (4)	C5A—H5AA	0.9300
N1B—C3B	1.336 (3)	C1B—C5B	1.400 (4)
N1B—H1BA	0.8600	C1B—C2B	1.404 (4)
C1—C1A	1.506 (4)	C2B—C3B	1.375 (4)
C1—C2	1.534 (4)	C3B—H3BA	0.9300
C1—H1A	0.9700	C4B—C5B	1.366 (4)
C1—H1B	0.9700	C4B—H4BA	0.9300
C2—C3	1.539 (4)	C5B—H5BA	0.9300
			
O2A—S1—O1A	114.62 (15)	C1B—C3—H3B	109.8
O2A—S1—O3A	112.98 (15)	C2—C3—H3B	109.8
O1A—S1—O3A	112.74 (14)	H3A—C3—H3B	108.3
O2A—S1—C2A	105.27 (13)	C5A—C1A—C2A	117.2 (3)
O1A—S1—C2A	105.09 (12)	C5A—C1A—C1	118.5 (2)
O3A—S1—C2A	105.00 (11)	C2A—C1A—C1	124.0 (2)
O1B—S2—O3B	115.5 (2)	C3A—C2A—C1A	119.8 (2)
O1B—S2—O2B	111.54 (16)	C3A—C2A—S1	116.9 (2)
O3B—S2—O2B	112.5 (2)	C1A—C2A—S1	123.30 (19)
O1B—S2—C2B	105.08 (15)	N1A—C3A—C2A	120.4 (3)
O3B—S2—C2B	106.43 (15)	N1A—C3A—H3AA	119.8
O2B—S2—C2B	104.76 (13)	C2A—C3A—H3AA	119.8
C3A—N1A—C4A	121.9 (3)	N1A—C4A—C5A	119.7 (3)
C3A—N1A—H1AA	119.1	N1A—C4A—H4AA	120.1
C4A—N1A—H1AA	119.1	C5A—C4A—H4AA	120.1
C4B—N1B—C3B	122.2 (2)	C4A—C5A—C1A	120.9 (3)
C4B—N1B—H1BA	118.9	C4A—C5A—H5AA	119.5
C3B—N1B—H1BA	118.9	C1A—C5A—H5AA	119.5
C1A—C1—C2	107.7 (2)	C5B—C1B—C2B	117.5 (2)
C1A—C1—H1A	110.2	C5B—C1B—C3	118.7 (3)
C2—C1—H1A	110.2	C2B—C1B—C3	123.8 (3)
C1A—C1—H1B	110.2	C3B—C2B—C1B	119.2 (2)
C2—C1—H1B	110.2	C3B—C2B—S2	116.38 (19)
H1A—C1—H1B	108.5	C1B—C2B—S2	124.4 (2)
C1—C2—C3	112.4 (2)	N1B—C3B—C2B	120.6 (2)
C1—C2—H2A	109.1	N1B—C3B—H3BA	119.7
C3—C2—H2A	109.1	C2B—C3B—H3BA	119.7
C1—C2—H2B	109.1	N1B—C4B—C5B	119.9 (3)
C3—C2—H2B	109.1	N1B—C4B—H4BA	120.1
H2A—C2—H2B	107.9	C5B—C4B—H4BA	120.1
C1B—C3—C2	109.3 (2)	C4B—C5B—C1B	120.6 (2)
C1B—C3—H3A	109.8	C4B—C5B—H5BA	119.7
C2—C3—H3A	109.8	C1B—C5B—H5BA	119.7
			
C1A—C1—C2—C3	178.2 (2)	C1—C1A—C5A—C4A	172.4 (3)
C1—C2—C3—C1B	−165.5 (3)	C2—C3—C1B—C5B	−94.3 (3)
C2—C1—C1A—C5A	−78.8 (3)	C2—C3—C1B—C2B	82.9 (3)
C2—C1—C1A—C2A	95.4 (3)	C5B—C1B—C2B—C3B	−0.1 (4)
C5A—C1A—C2A—C3A	2.3 (4)	C3—C1B—C2B—C3B	−177.4 (3)
C1—C1A—C2A—C3A	−172.0 (2)	C5B—C1B—C2B—S2	177.4 (2)
C5A—C1A—C2A—S1	−178.4 (2)	C3—C1B—C2B—S2	0.2 (4)
C1—C1A—C2A—S1	7.3 (4)	O1B—S2—C2B—C3B	−15.5 (3)
O2A—S1—C2A—C3A	9.9 (2)	O3B—S2—C2B—C3B	−138.4 (3)
O1A—S1—C2A—C3A	−111.5 (2)	O2B—S2—C2B—C3B	102.2 (2)
O3A—S1—C2A—C3A	129.4 (2)	O1B—S2—C2B—C1B	166.9 (2)
O2A—S1—C2A—C1A	−169.4 (2)	O3B—S2—C2B—C1B	44.0 (3)
O1A—S1—C2A—C1A	69.2 (2)	O2B—S2—C2B—C1B	−75.4 (2)
O3A—S1—C2A—C1A	−50.0 (2)	C4B—N1B—C3B—C2B	−0.1 (4)
C4A—N1A—C3A—C2A	−2.3 (5)	C1B—C2B—C3B—N1B	0.7 (4)
C1A—C2A—C3A—N1A	−0.2 (4)	S2—C2B—C3B—N1B	−177.00 (19)
S1—C2A—C3A—N1A	−179.5 (2)	C3B—N1B—C4B—C5B	−1.0 (4)
C3A—N1A—C4A—C5A	2.4 (5)	N1B—C4B—C5B—C1B	1.6 (4)
N1A—C4A—C5A—C1A	−0.1 (5)	C2B—C1B—C5B—C4B	−1.0 (4)
C2A—C1A—C5A—C4A	−2.2 (5)	C3—C1B—C5B—C4B	176.4 (3)

### Hydrogen-bond geometry (Å, °)

**Table:**

*D*—H···*A*	*D*—H	H···*A*	*D*···*A*	*D*—H···*A*
N1A—H1AA···O3A^i^	0.86	1.88	2.706 (3)	159
N1A—H1AA···S1^i^	0.86	2.79	3.633 (3)	165
N1B—H1BA···O2B^ii^	0.86	1.85	2.710 (3)	176
N1B—H1BA···S2^ii^	0.86	2.85	3.655 (2)	158
C3B—H3BA···O1A^iii^	0.93	2.44	3.008 (3)	119
C4B—H4BA···O2A^iv^	0.93	2.50	3.035 (3)	117
C5B—H5BA···O1B^v^	0.93	2.58	3.416 (4)	150

Symmetry codes: (i) −*x*+1, *y*+1/2, −*z*+1/2; (ii) −*x*+1, *y*−1/2, −*z*+3/2; (iii) *x*+1/2, −*y*+3/2, −*z*+1; (iv) −*x*+1, *y*−1/2, −*z*+1/2; (v) −*x*+1/2, −*y*+1, *z*−1/2.
